# Correction: Patsouris et al. Advances in Innovative Surgical Implant Manufacturing for Hernia Repair and Soft Tissue Reconstruction. *Bioengineering* 2025, *12*, 1182

**DOI:** 10.3390/bioengineering13040481

**Published:** 2026-04-21

**Authors:** Stavros Patsouris, Panagiotis Mallis, Efstathios Michalopoulos, Nefeli Papadopoulou, Michalis Katsimpoulas, Nikolaos Nikiteas

**Affiliations:** 1 School of Medicine, National and Kapodistrian University of Athens, Mikras Asias 75, 115 27 Athens, Greece; 2 Hellenic Cord Blood Bank, Biomedical Research Foundation Academy of Athens, Soranou tou Efesiou 4, 115 27 Athens, Greece; pmallis@bioacademy.gr (P.M.); smichal@bioacademy.gr (E.M.); 3 Independent Researcher, 106 80 Athens, Greece; nefpap92@gmail.com; 4 Center for Clinical, Experimental Surgery and Translational Research, Biomedical Research Foundation of the Academy of Athens, 115 27 Athens, Greece; mkatsiboulas@bioacademy.gr; 5 Second Propaedeutic Department of Surgery, School of Medicine, National and Kapodistrian University of Athens, 115 27 Athens, Greece; nikolaos.nikiteas@gmail.com

**Revisions to Authorship and Affiliation**

In the published publication [1], the order of the authors was not correct. In the corrected version, Dr. Nikolaos Nikiteas is listed as the last author.

Additionally, the affiliation of Dr. Nikolaos Nikiteas was revised as follows: Second Propaedeutic Department of Surgery, School of Medicine, National and Kapodistrian University of Athens, 115 27 Athens, Greece.

The requested changes are necessary for formal academic and institutional reasons related to the doctoral research of Dr. Stavros Patsouris, which was conducted under the supervision of Professor Nikiteas at the 2nd Propaedeutic Department of Surgery, School of Medicine, National and Kapodistrian University of Athens.

All co-authors have been informed of the requested correction and provided their full agreement and approval. The authors state that the scientific conclusions are unaffected. This correction was approved by the Academic Editor. The original publication has also been updated.
